# Pathophysiology of Post-COVID syndromes: a new perspective

**DOI:** 10.1186/s12985-022-01891-2

**Published:** 2022-10-09

**Authors:** Gaber El-Saber Batiha, Hayder M. Al-kuraishy, Ali I. Al-Gareeb, Nermeen N. Welson

**Affiliations:** 1 Department of Pharmacology and Therapeutics, Faculty of Veterinary Medicine, Damanhour University, Damanhour, Al Beheira, 22511 Egypt; 2 Department of Clinical Pharmacology and Medicine, College of Medicine, ALmustansiriyia University, Baghdad, Iraq; 3 Department of Forensic Medicine and Clinical Toxicology, Faculty of Medicine, Beni-Suef University, Beni Suef, 62511 Egypt

**Keywords:** COVID-19, Post-COVID syndrome, Mast cell activation syndrome, Pathogenesis

## Abstract

Most COVID-19 patients recovered with low mortality; however, some patients experienced long-term symptoms described as “long-COVID” or “Post-COVID syndrome” (PCS). Patients may have persisting symptoms for weeks after acute SARS-CoV-2 infection, including dyspnea, fatigue, myalgia, insomnia, cognitive and olfactory disorders. These symptoms may last for months in some patients. PCS may progress in association with the development of mast cell activation syndrome (MCAS), which is a distinct kind of mast cell activation disorder, characterized by hyper-activation of mast cells with inappropriate and excessive release of chemical mediators. COVID-19 survivors, mainly women, and patients with persistent severe fatigue for 10 weeks after recovery with a history of neuropsychiatric disorders are more prone to develop PCS. High D-dimer levels and blood urea nitrogen were observed to be risk factors associated with pulmonary dysfunction in COVID-19 survivors 3 months post-hospital discharge with the development of PCS. PCS has systemic manifestations that resolve with time with no further complications. However, the final outcomes of PCS are chiefly unknown. Persistence of inflammatory reactions, autoimmune mimicry, and reactivation of pathogens together with host microbiome alterations may contribute to the development of PCS. The deregulated release of inflammatory mediators in MCAS produces extraordinary symptoms in patients with PCS. The development of MCAS during the course of SARS-CoV-2 infection is correlated to COVID-19 severity and the development of PCS. Therefore, MCAS is treated by antihistamines, inhibition of synthesis of mediators, inhibition of mediator release, and inhibition of degranulation of mast cells.

## Key summary points

- Post-COVID (PCS) syndrome may progress in association with the development of mast cell activation syndrome (MCAS).
- High D-dimer levels and blood urea nitrogen were observed to be risk factors associated with pulmonary dysfunction in COVID-19 survivors 3 months post-hospital discharge with the development of PCS.
- Persistence of inflammatory reactions, autoimmune mimicry, and reactivation of pathogens together with host microbiome alterations may contribute to the development of PCS.
- MCAS is treated by antihistamines, inhibition of synthesis of mediators, inhibition of mediator release, and inhibition of degranulation of mast cells.

## Introduction

The current devastating coronavirus disease 2019 (COVID-19) pandemic leads to a worldwide impact with high morbidity and relative mortality in high-risk group patients [1]. The COVID-19 is caused by a novel single-strand RNA virus named the severe acute respiratory syndrome coronavirus 2 (SARS-CoV-2) [2, 3]. The clinical feature of COVID-19 is asymptomatic or mild flu-like illness in the majority of cases [4, 5]. However, about 15% of COVID-19 patients experienced features of acute viral pneumonia due to the propagation of acute lung injury (ALI). Approximately 5% of COVID-19 patients need intensive care unit admission and ventilation support due to the development of acute respiratory distress syndrome (ARDS) [6, 7].

SARS-CoV-2 exploits and bind specific cell membrane receptor named angiotensin converting enzyme 2 (ACE2) [8]. ACE2 is highly distributed and expressed in many organs, including the intestine, kidney, lung, brain, heart, testis, and some immune cells [8, 9]. Severe SARS-CoV-2 infection induces an exaggeration of immune response and release of pro-inflammatory cytokines with the progression of hypercytokinemia and cytokine storm [10, 11].

Most COVID-19 patients recovered with a low mortality rate of 3–5%. However, some patients experienced long-term symptoms described as long-COVID or Post-COVID syndrome (PCS) [12, 13]. PCS was initially identified by a scientific group in early 2020 following a strict survey of long-term symptoms in recovered COVID-19 patients. They found that COVID-19 patients may have persisting symptoms for weeks after acute SARS-CoV-2 infection, including dyspnea, fatigue, myalgia, insomnia, cognitive and olfactory disorders [14, 15]. However, these symptoms may last for months in some patients, which may affect their daily activities [16, 17].

**Fig. 1:**
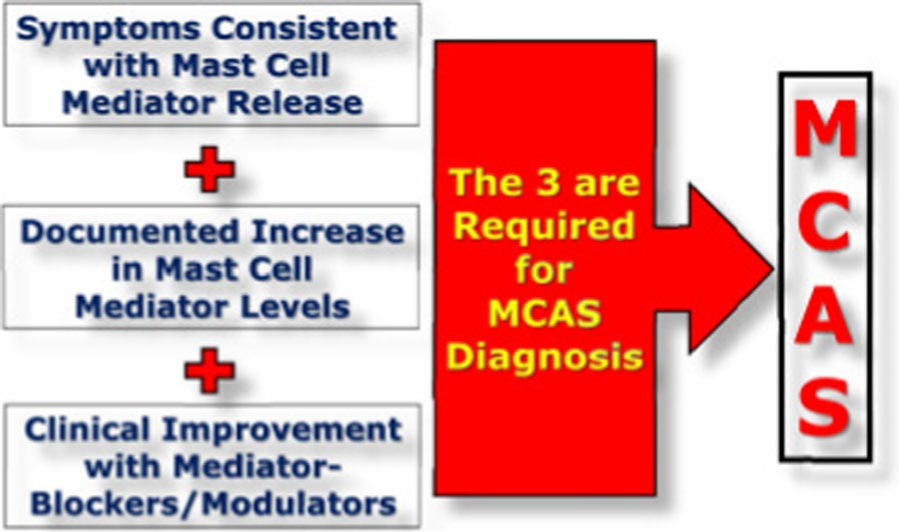
Diagnostic criteria of mast cell activation syndrome

It has been shown that PCS may progress in association with the development of mast cell activation syndrome (MCAS) [18]. MCAS is a distinct kind of mast cell activation disorder characterized by hyper-activation of mast cells with excessive and inappropriate release of chemical mediators [19]. The primary manifestations of MCAS are cardio-pulmonary, gastrointestinal, dermatological, and neurological problems [19]. MCAS differs from mastocytosis, which is characterized by an increase in the number and abnormal shape of mast cells. In MCAS, the number and shape of mast cells are normal [20]. However, symptoms and presentations of MCAS and systemic mastocytosis are intermingled with the development of extensive tissue damage and can be treated by receptor blockade of relevant mediators including histamine, leukotrienes, and prostaglandin, or by inhibiting mediator synthesis [21]. Diagnosis of MCAS depends on the presence of symptoms consistent with mediator release, increased mast cell mediators and clinical improvement with mediator blockers [20] (Fig. 1). Therefore, this critical review aimed to find the crucial association between PCS and MCAS with appropriate therapeutic modalities.

### Post-COVID syndrome

Imam Ali, cousin of the Messenger of God in the sixth century, said: “And considers what has passed for what is to come.” Now, what passed, I think, will appear.

PCS was initially defined in relation to post-acute COVID-19 as persistence symptoms for > 3 weeks from the onset of COVID-19 or chronic COVID-19 as persistence symptoms for > 12 weeks from the onset of COVID-19 [22, 23]. Yong defined PCS or long-term COVID-19 as symptoms that persist for more than 3 months after the onset of COVID-19 [24, 25]. However, different studies reported that patients with PCS had persistence symptoms in contrasting frequencies and durations of COVID-19 survivors. This might be due to dissimilar characteristics of sample size and methods for data collection [26–28]. As a result of large studies, there are different descriptions of PCS according to the duration of symptoms (Table 1).

**Table 1:** Descriptions of Post-COVID syndrome

Duration of symptoms	Description/terms	References
> 3 months	Post-COVID syndrome	[22, 29, 30]
	Long COVID	
	Chronic COVID-19	
1–3 months	On-going COVID-19	[22]
	Post-acute COVID-19	
> 24 weeks	Persistent post-COVID symptoms	[31]
12–24 weeks	Long post-COVID symptoms	[32]
5–12 weeks	Acute post-COVID symptoms	[33]
> 4 weeks (from symptoms onset)	Post-acute COVID-19 syndrome	[34]
> 4 weeks (from diagnosis time)	Long-COVID	[35–37]
	Long-haulers	
	Late sequelae of SARS-CoV-2 infection	
> 2 months	Long-COVID	[38]
> 100 days	Long-haul COVID	[39]

The clinical presentations of PCS are still elusive, comprising different phenotypes and subtypes [40]. Of interest, PCS may develop in patients with mild-moderate COVID-19 and even in asymptomatic patients [41, 42]. As well, PCS may develop in asymptomatic children with COVID-19, resulting in persistent dyspnea and fatigue [43]. PCS may be similar to post-viral syndrome as in SARS and Middle East Respiratory Syndrome (MERS) [44, 45]. Prolonged signs and symptoms could be evident for 7–15 years following previous SARS, mainly in younger age groups [46]. Therefore, PCS may persist for years following the onset of COVID-19.

### Risk factors for Post-COVID syndrome

It has been shown that COVID-19 survivors, mainly women, and patients with persistent severe fatigue for 10 weeks after recovery with a history of neuropsychiatric disorders are more prone to developing PCS [47]. Thus, female sex is regarded as a potential risk factor for the development of PSC due to higher immunological response and hormonal changes [48]. However, some studies found no gender difference in the risk of developing PCS after an acute SARS-CoV-2 infection [49, 50]. Furthermore, co-morbidities, advanced age, and the severity of the initial disease are associated with an increased risk of developing PCS [51]. Staven et al. found that follow-up of COVID-19 survivors after 1–6 months of their first SARS-CoV-2 infection revealed the presence of 10 symptoms during the initial acute SARS-CoV-2 infection increased their risk for development of PCS [52, 53]. In addition, patients with severe initial SARS-CoV-2 infection are regarded as a high-risk group for the development of PCS [54]. Townsend et al. observed that persistent fatigue and development of PCS are common and independent of initial COVID-19 severity [47, 55]. As well, COVID-19 survivors with initial SARS-CoV-2 infection who needed hospitalization and ICU admission with a requirement for supported ventilation were highly predisposed to develop PCS 3 months later [56]. Of note, acute disease-induced extensive tissue injury may lead to the development of post-intensive care syndrome, which is characterized by long-term neuropsychiatric disorders and physical impairment [57]. Stam et al. [58] give a call for risk of development of post-intensive care syndrome in COVID-19 patients admitted in ICU. These findings suggest that COVID-19 could have an additive impact on the severity of post-intensive care syndrome and vice versa.

On the other hand, biomarkers of COVID-19 severity are linked with the progression of PCS. For example, high D-dimer level and blood urea nitrogen were observed to be risk factors associated with pulmonary dysfunction in COVID-19 survivors 3 months post-hospital discharge with the development of PCS [59, 60]. Similarly, high levels of CRP, D-dimer, and IL-6 are related to pulmonary dysfunction and the advancement of PCS [61]. In addition, Raman et al. revealed that high systemic biomarkers of inflammation and lymphopenia are also linked with radiological lesions of various organs within 3 months in post-discharge COVID-19 survivors [62]. Surprisingly, high troponin levels are associated with the development of fatigue, whereas lymphopenia is associated with an increased risk of tachycardia in PCS patients [63]. Therefore, persistent symptoms in COVID-19 survivors must be correlated with inflammatory biomarkers and lymphocyte counts to predict the development of PCS.

### Clinical presentation of Post-COVID syndrome

PCS includes a plethora of conditions and symptoms, and the incidence of explicit symptoms may vary according to the severity, duration, and nature of acute infection [44]. Fatigue represents the most common symptom of PCS and is found in 17–72% of critically ill COVID-19 patients [64]. Respiratory symptoms are more common in PCS patients, including chest pain (22%), dyspnea (10–40%), and exercise intolerance (10–40%), though worsening dyspnea in PCS patients has been reported to exaggerate by up to 65% during ICU hospitalization [65]. Arrhythmias, postural hypotension, and persistently high blood pressure with hypertension develop due to endothelial dysfunction and cardiac injury [66]. Gastrointestinal symptoms such as nausea, vomiting, diarrhea, change bowel habit, appetite disorders may remain in 30% of COVID-19 patients for more than two months following hospital discharge [67, 68].

Furthermore, patients with PCS may have neuropsychiatric disorders such as olfactory/gustatory dysfunction, which occurs in 9–11% of patients and can last 6–8 months after mild COVID-19 [69]. As well, PCS patients may experience anxiety in 26% and depression in 40% of their cases within or after six months from the onset of acute COVID-19 [70, 71]. Psychiatric complications in PCS patients, including aggression, cognitive deficit, reduction of social activity, and obsessive–compulsive disorders, have been reported [72]. Furthermore, post-traumatic stress disorders have been shown to be found in recovered COVID-19 patients by up to 43% [73, 74]. Other neurological disorders observed in PCS patients are Guillain–Barre syndrome, seizures, ischemic stroke, cerebral vasculitis, and transverse myelitis [75].

These findings suggest that PCS has systemic manifestations which might be mild or severe. However, Moreno-Perez et al. illustrated that the majority of persisting symptoms in PCS are resolved with time with no further complications [76]. Though the final results of PCS are largely unknown.

### Category of Post-COVID syndrome

It has been proposed that PCS symptoms are classified into residual symptoms (persist after recovery from acute SARS-CoV-2 infection), organ dysfunction symptoms (persist following recovery) and new symptoms (developed after mild SARS-CoV-2 infection) [27]. However, PCS was classified according to the onset, type, and duration of symptoms into 5 types: [22] (Table 2).

**Table 2:** Types of Post-COVID syndrome

Types	Initial symptoms	Duration of symptoms	Quiescence period	Delayed symptoms
Type I	Variable	Variable	Negative	Negative
Type II	Mild	> 6 weeks	Negative	Negative
Type IIIA	Mild	3–6 months	Present	Negative
Type IIIB	Mild	> 6 months	Present	…………
Type IVA	Negative	Variable	Negative	> 3 months
Type IVB	Negative	Variable	Negative	> 6 months
Type V	Negative	…………	…………	Present

Type I: PCS patients have varying durations of recovery, and the symptoms are linked to the severity and complications of the SARS-CoV-2 infection.

Type II: PCS patients have persisting symptoms for 6 weeks from the onset of the SARS-CoV-2 infection.

Type III: PCS patients show a recovery period followed by a re-appearance of symptoms.

Type IIIA: symptoms persist for 3 months. Type IIIB: symptoms persist for 6 months.

Type IV: PCS patients were initially asymptomatic at the time of diagnosis but later became symptomatic.

Type IVA: become symptomatic within 1–3 months, Type IVB: become symptomatic after 3 months.

Type V: PCS patients were initially asymptomatic at time of diagnosis and die within 12 months.

**Fig. 2:**
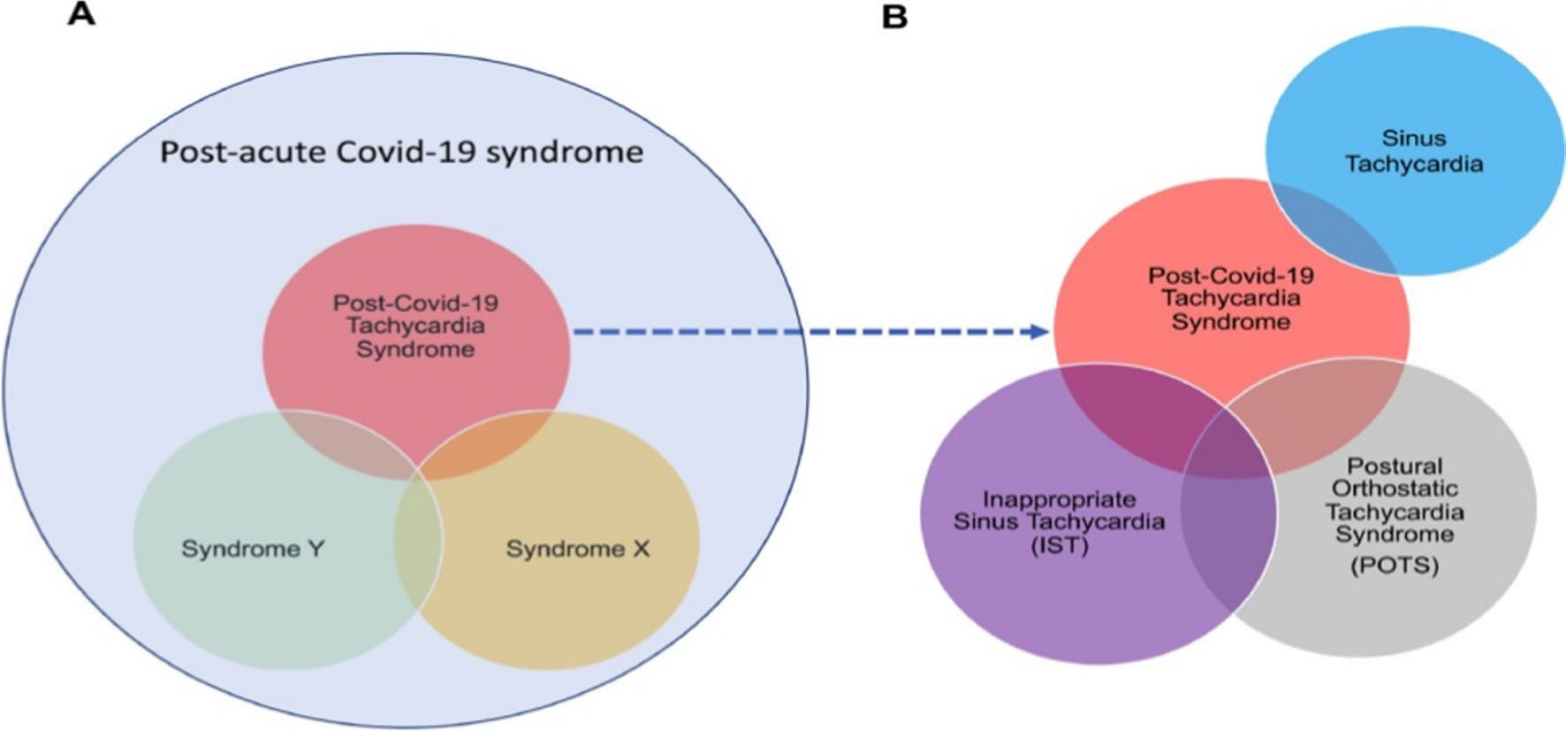
Post-COVID tachycardia syndrome [77]

Of interest, post-COVID POTS is regarded as a distinct type of PCS characterized by sinus tachycardia, postural tachycardia, and inappropriate sinus tachycardia (Fig. 2).

### Pathogenesis of Post-COVID syndrome

Systemic inflammatory response syndrome (SIRS) could be the potential cause for the development of organ dysfunction and tissue injury in PCS. Due to the development of an exaggerated immune response and a high pro-inflammatory response in COVID-19, a counter-balanced anti-inflammatory response is developed, leading to a state of immunosuppression to maintain immunological homeostasis [78, 79]. However, prolonged immunosuppression may cause propagation catabolism syndrome and the development of PCS [80]. It has been reported that post-septic patients are susceptible to latent viral infections and reactivations [81] and a relapse of SARS-CoV-2 infection in recovered COVID-19 with the development of secondary infections [82, 83]. Further, Russell et al., exemplified that transforming growth factor β (TGF-β) which is an immunosuppressive, profibrotic, and anti-inflammatory cytokine, is increased during and after SARS-CoV-2 infection to dampen an exaggerated pro-inflammatory response [84]. TGF-β is associated with the development of pulmonary interstitial fibrosis in COVID-19 patients [85]. As a result, targeting TGF-β in COVID-19 patients may have a therapeutic value in reducing the fibrotic changes in PCS [86].

**Fig. 3:**
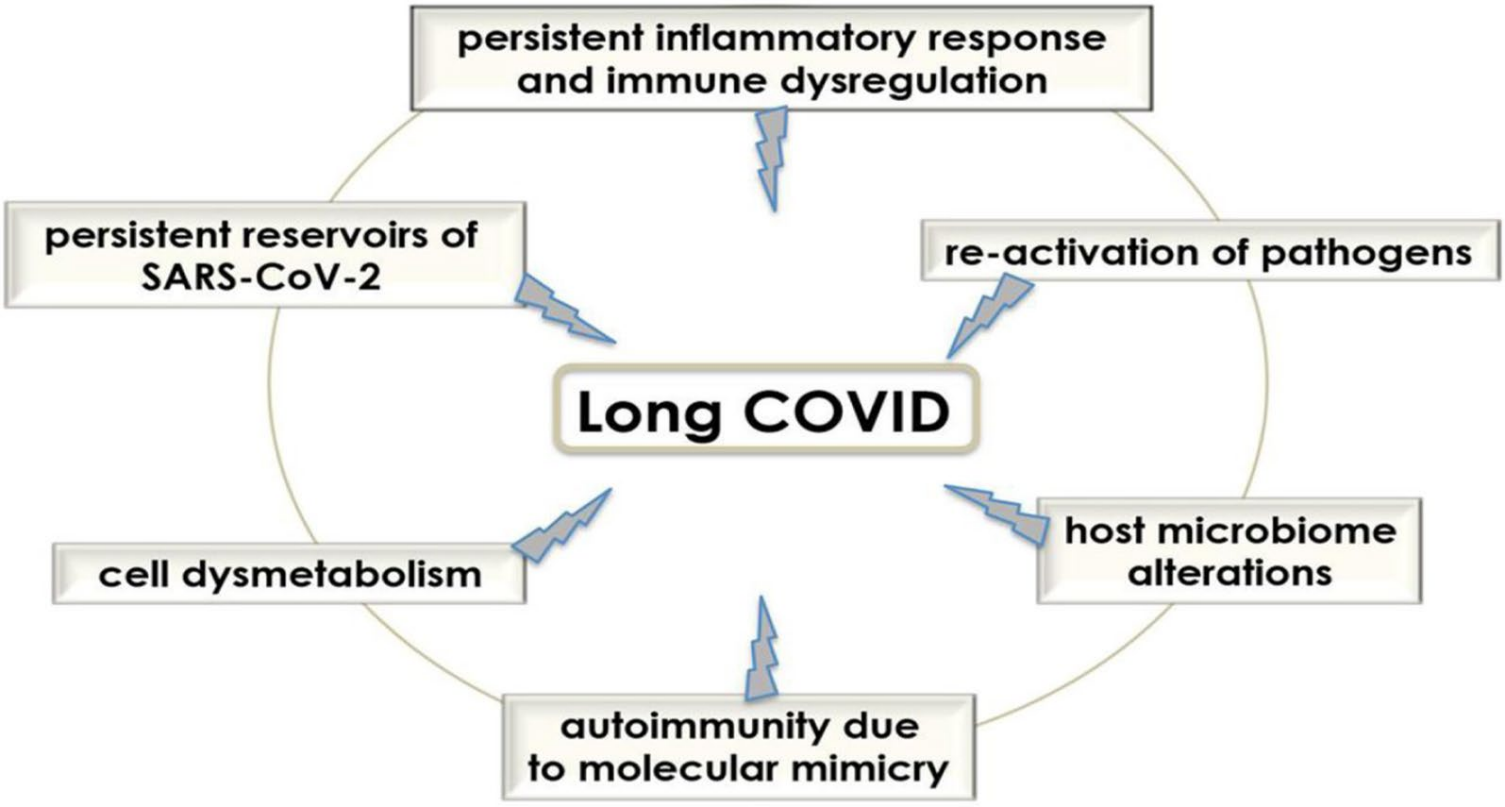
Pathogenesis of Post-COVID syndrome

Several studies [87, 88] have reported the persistence of subclinical and/or symptomatic SARS-CoV-2 infection for up to 3 months after infection. Several studies [89, 90] have found SARS-CoV-2 shedding in both lungs for 4 months and in the GI tract for 2 months. Persistence SARS-CoV-2 infection triggers long-term immune stimulation and development of PCS. Persistent SARS-CoV-2 infection activates autoreactive T cells through presentation of antigens by antigen-presenting cells as a bystander effect in MIS syndrome [91]. Of interest, MIS may develop in children and adults within 2–6 weeks and is correlated with pro-inflammatory cytokine levels, but it was not related to the severity of the initial SARS-CoV-2 infection [91]. The delayed onset of MIS following SARS-CoV-2 infection may be due to the deregulation of adaptive immune responses [92]. The stimulation of T cell-mediated immunity in PCS is evident by the development of autoimmune-mediated thyroid dysfunction [93]. Likewise, B cell activation and the production of antiphospholipid autoantibodies were detected in 52% of PCS patients [94]. Similarly, autoantibodies were detected in up to 50% of COVID-19 patients and PCS, indicating a link with the development of autoimmune diseases including systemic lupus erythematosus [95]. Prolonged lymphopenia is associated with PCS patients' chronic immune activation and hyperinflammation [76]. It has been shown that lymphopenia is related to persistent SARS-CoV-2 infection, abnormal immune response, and persistent COVID-19 patient’s symptoms in PCS [96]. Taken together, immune dysregulation, persistence of inflammatory reactions, autoimmune mimicry, and reactivation of pathogens together with host microbiome alterations may contribute to the development of PCS (Fig. 3).

**Fig. 4:**
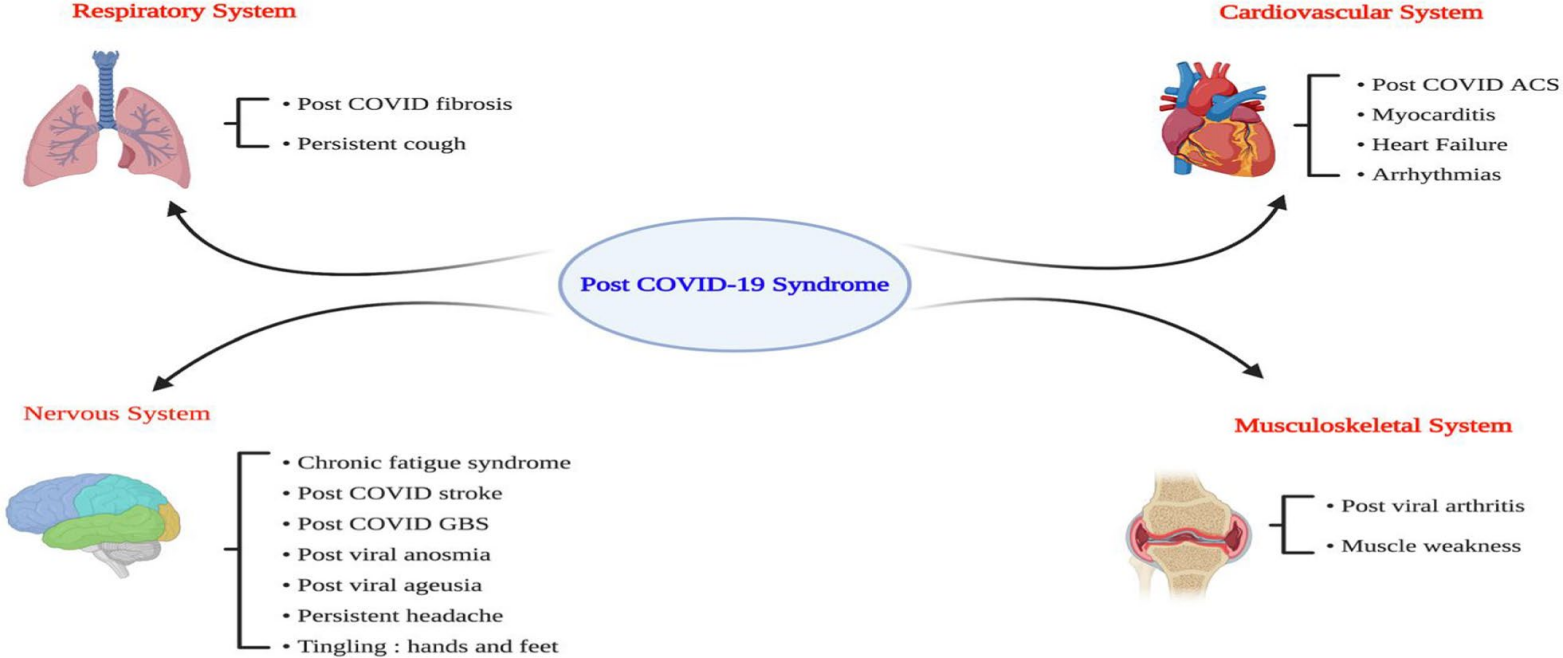
Systemic effects of Post-COVID syndrome

Therefore, unresolved lymphopenia and protracted high pro-inflammatory cytokine levels are linked with the development of headaches, joint pain, and fatigue, the cardinal symptoms of PCS [44]. As well, fatigue is also related to the development of cerebral hypoperfusion, muscle channelopathy, and dysautonomia [44]. In turn, PCS leads to systemic effects involving the cardiovascular, respiratory, nervous system, and musculoskeletal systems (Fig. 4).

### Pulmonary complications

Pulmonary complications Organic or functional impairments have been reported in SARS-CoV-2 survivor COVID-19 patients following acute pneumonia [97]. Most COVID-19 patients experience mild-moderate respiratory complications, and about 5% of them develop ARDS [6]. It has been reported that ARDS has three pathological phases (exudative, proliferative, and fibrotic) [98].

In a follow-up study of 55 survivor COVID-19 patients, symptoms related to SARS-CoV-2 infection were detected in 35 patients, and radiological abnormalities in 39 patients that correlated with high blood urea nitrogen (OR = 7.14, 95% CI = 1.03–49.21, *P* = 0.04). Functional pulmonary disorders were found in 14 patients that correlated with D-dimer (OR = 1.066, 95% CI = 1.006–1.129, *P* = 0.03) [97]. In 42% of the COVID-19 patients' survivors 3 months after hospital discharge, there was a significant reduction in lung diffusion capacity [99]. Remarkably, persistent symptoms and pulmonary radiological changes may persist for 6 months in 50% of COVID-19 patients' survivors [100].

Of note, radiological abnormalities and lung fibrosis may persist up to 6 months following an acute SARS-CoV-2 infection [101]. Besides, pulmonary dysfunction with impairment of gas exchange was detected in patients with moderate COVID-19 even with a normal lung computed tomography (CT) scan [102]. Likewise, maximal aerobic capacity was reduced in follow-up COVID-19 patients compared with controls [103].

Disruption of endothelial integrity and alveolar injury due to the release of pro-inflammatory cytokines and infiltration of immune cells are highly evident in the exudative phase [98]. In the fibro-proliferative phase, deposition of collagen and fibronectin in the alveolar space occurred due to accumulation of fibrocytes, myofibroblasts, and fibroblasts [104]. These pathological changes trigger the release of TGF-β, inhibition of the collagenase enzyme, and further deposition of collagen [104]. Most of the survivor patients from COVID-19 with ARDS managed by mechanical ventilation develop lung fibrosis and pulmonary dysfunction [105]. It has been shown that survivors of ARDS may have exercise intolerance with a profound reduction in life quality for about 5 years [106].

Indeed, imbalance between helper T cells and regulatory T cells as well as augmentation of CD8 + T cells were observed in PCS, resulting in the progression of an autoimmune response that persisted for a long time [107]. An imbalance of T cell immune response was detected to be associated with pulmonary complications, as mature T cells have the ability to produce granzyme B, which is elevated in COVID-19 survivors [108].

**Fig. 5:**
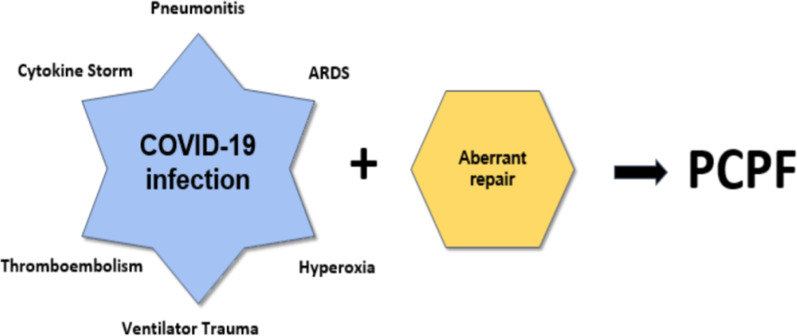
COVID-19 and the risk of Post-COVID pulmonary fibrosis (PCPF)

Overall, pathological changes, including cytokine storm, ALI, ARDS, ventilation injury, hypoxia, and hyperoxia, may interact together to induce an aberrant repair with the development of post-COVID pulmonary fibrosis (Fig. 5).

These findings suggest that lung fibrosis/scarring could be the possible causes of chronic cough and dyspnea in patients with PCS. However, results from a cohort-prospective study revealed that respiratory symptoms may persist in patients with PCS even with enhancement of pulmonary function and resolution of radiological abnormalities [109]. This verdict suggests that other pathophysiological mechanisms might be responsible for dyspnea in patients with PCS.

### Neurological complications

In general, SARS-CoV-2 infection is associated with neurological manifestations and complications like dysgeusia and anosmia due to the neurotropic effect of SARS-CoV-2 [110]. It has been shown that neurological manifestations are present in 36.4% of COVID-19 patients, including central and peripheral neurological complications as well as skeletal muscle disorders [111, 112]. The most common neurological symptoms in COVID-19 are dizziness (16.8%), headache (13.1%), and fatigue (13.0%) [113]. In addition, stroke, seizure, ataxia, and confusion were also documented as central neurological manifestations/complications in COVID-19 [113].

Of interest, fatigue in PCS is developed due to autoantibodies against muscarinic and adrenergic receptors, resulting in an association between fatigue and dysautonomia [114]. An association between anxiety and fatigue proposed the development of Myalgic Encephalomyelitis/Chronic Fatigue Syndrome (ME/CFS), which is characterized by chronic fatigue, cognitive impairment, dysautonomia, and endocrinopathies [115]. It has been reported that ME/CFS is linked with the production of autoantibodies that injure phospholipids, gangliosides, and 5-hydroxytryptamine [116]. ME/CFS is more common in women, and has been reported to develop after 6 months of the initial SARS-CoV-2 infection [117].

Similarly, neuropsychiatric disorders including depression, psychosis, and anxiety have been reported following COVID-19 [118]. Mazza and colleagues reported that depression and anxiety in COVID-19 survivors were correlated with high inflammatory burden [119], since hyper-active immune response and neuroinflammation increase the risk of development of neuropsychiatric complications in COVID-19 [118]. In an MRI-based study for the assessment of neurological changes in COVID-19 survivors 3 months following discharge, there were significant structural changes which interrelated with prolonged neurological symptoms such as cognitive deficits and anosmia [120]. In a prospective study involving 60 COVID-19 survivors compared to 39 matched controls, there were neurological dysfunctions in 55% of the COVID-19 survivors compared to healthy controls (*P* < 0.05). Some of these structural brain changes were correlated with high LDH levels [120]. Therefore, the long-term effects of SARS-CoV-2 infection, even a mild-moderate one, may affect functional and micro-structural brain integrity, resulting in neurological consequences in patients with PCS.

Furthermore, Paterson et al., illustrated a high frequency of acute disseminated encephalomyelitis in COVID-19 survivors that was not correlated with the initial severity of COVID-19 [121]. This finding proposes that SARS-CoV-2 infection, regardless of its severity, may cause long-term neurological complications, and this may explain neuropsychiatric manifestations in patients with PCS.

In addition, delirium was reported in hospitalized patients with severe COVID-19 and may be present in patients with PCS [122]. It has been observed that early presentation of delirium in SARS-CoV-2 infection may predict the development of cognitive dysfunction, mainly in elderly survivors [123]. A meta-analysis study of 20 studies revealed that delirium symptoms in COVID-19 patients at the time of admission were linked with poor neurological outcomes (OR = 2.36, 95% CI = 1.80–3.09, *P* < 0.00001) and high mortality [123].

Rogers and colleagues revealed that neuropsychiatric symptoms in the acute phase of SARS-CoV-2 infection may lead to fatigue, cognitive impairment, and other neuropsychiatric squeals due to brain dysfunction [124]. Besides, a retrospective cohort study involving 236,379 COVID-19 survivors six months following acute SARS-CoV-2 infection illustrated that 56% of COVID-19 survivors developed various neuropsychiatric spectrums, mainly with ICU admission [125].

Indeed, brainstem injury in acute SARS-CoV-2 infection may cause cardio-respiratory dysfunction due to injury to respiratory and vasomotor centers [126]. Brainstem dysfunction may persist for a long time after acute SARS-CoV-2 infection, leading to dyspnea and neurological dysfunction in patients with PCS [127]. Higher expression of ACE2 in the brainstem increases its susceptibility to SARS-CoV-2 neurotropism and subsequent inflammatory reaction-induced dysfunction [127]. In this state, post-mortem studies demonstrated that SARS-CoV-2 proteins and genes were detected in COVID-19 victims [128, 129].

The underlying mechanism of neuropsychiatric disorders in COVID-19 could be due to cytokine storm-induced disruption of the blood brain barrier (BBB), neuroinflammation, and peripheral neuronal injury, or due to direct SARS-CoV-2 neurotropism [130]. Notably, exaggerated inflammatory response with high TGF-β might be the proposed mechanism for the development of neuropsychiatric and other neurological disorders in COVID-19 [131]. The underlying proposed mechanism for neurological involvement in COVID-19 could be linked with the development of demyelination disorders since previous coronavirus infections have caused neurodegeneration and demyelination [132]. As well, the high distribution of ACE2 in specific brain regions such as the substantia nigra and limbic system may increase the interaction between SARS-CoV-2 and neurons, with subsequent neurological complications [133, 134].

These observations suggest that SARS-CoV-2 infections may lead to long-term neurological complications which participate in the development of PCS.

**Fig. 6:**
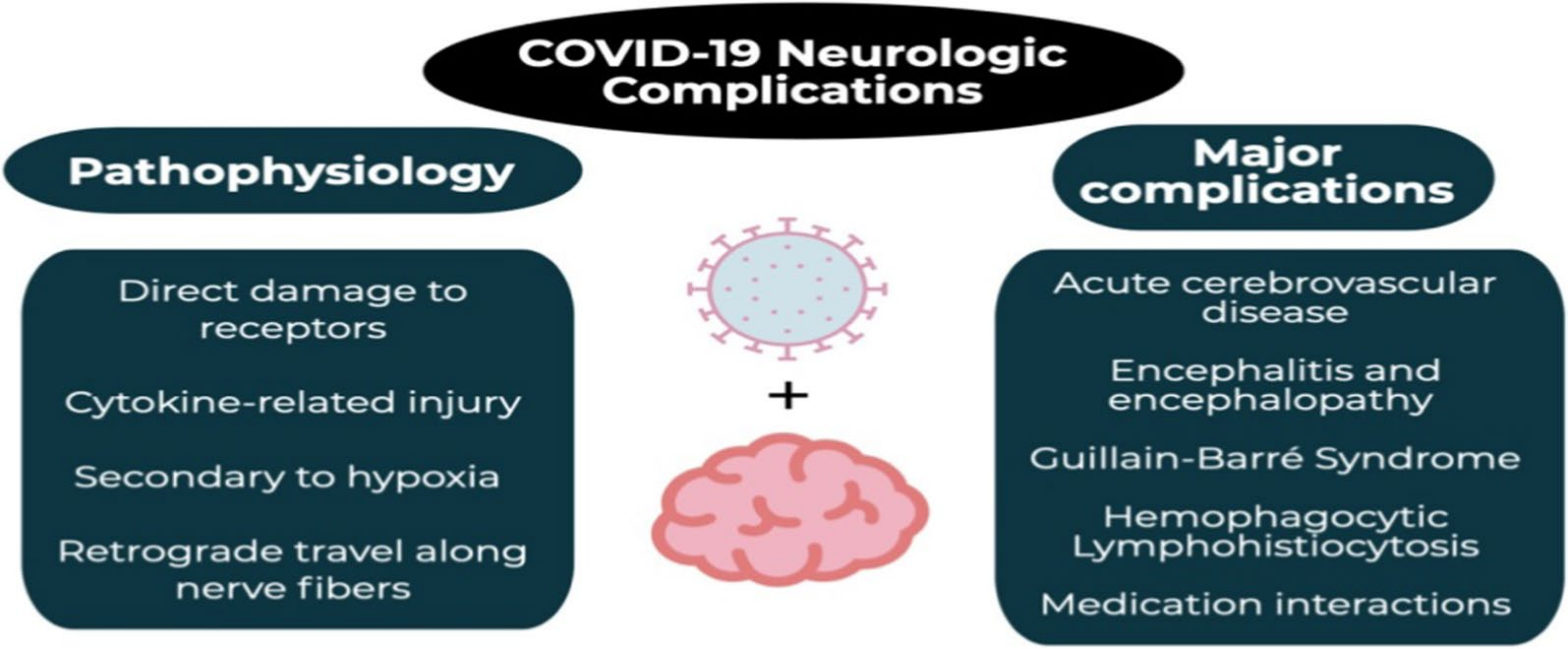
Post-COVID syndrome and neurological complications

Neurological manifestations in PCS, mainly fatigue and cognitive deficits, are related to the presence of autoantibodies against β2 adrenoceptors, α1-adrenoceptors, AT1R, MAS receptors, muscarinic type 2 receptors, and nociceptin-like opioid receptors, leading to dysautonomia, POTS, and other neurological dysfunctions [135]. The underlying pathophysiology of neurological manifestations and complications in COVID-19 with the possibility of development of PCS is complex (Fig. 6).

### Cardiovascular complications

It has been shown that cardiac fibrosis, endothelial dysfunction, and other cardiovascular complications are evident in patients with PCS [136]. In severe SARS-CoV-2 infections, myocarditis and acute cardiomyocyte injury may lead to the development of heart failure in COVID-19 patients [137]. The potential mechanisms of cardiomyocyte injury in COVID-19 include direct cardiomyocyte injury by SARS-CoV-2, hypoxia, cytokine storm, increased pulmonary vascular-induced pulmonary hypertension, augmentation of RAS, endothelial dysfunction, coagulopathy, and acute coronary syndrome [137, 138]. These changes lead to myocardial remodeling and cardiac fibrosis through the induction of expression and upregulation of TGF-β [139]. Nalbandian et al., observed that cardiac fibrosis and resultant cardiomyopathy and heart failure from SARS-CoV-2 infection in COVID-19 survivors can lead to tachyarrhythmia and exertional dyspnea in patients with PCS [140].

Indeed, SARS-CoV-2-induced autonomic dysfunction and dysautonomia in COVID-19 survivors can cause sinus tachycardia and postural orthostatic tachycardia syndrome (POTS) [12, 141]. POTS is more common in women and is caused by an autoimmune reaction to SARS-CoV-2 infection [142].Furthermore, hypercytokinemia's exaggerated catecholaminergic state may cause cardiac action potential prolongation by modulating the expression of cardiomyocyte ion channels [143].

In a study of 100 COVID-19 survivors, Puntman et al., discovered that 60% of them have myocarditis and 78% have other cardiac abnormalities [144]. Interestingly, these cardiac pathological changes were not related to the initial severity of COVID-19 [144]. A longitudinal multicenter echocardiographic study involving COVID-19 survivors three months after previous COVID-19 pneumonia showed that 29% of them had some degree of myocardial remodeling [145]. Furthermore, even in mild or asymptomatic patients, asymptomatic COVID-19 may be associated with myocardial inflammation, as evidenced by cardiac magnetic resonance in athletes recovered from SARS-CoV-2 infection [146].

Long-term cardiac complications COVID-19 survivors need further long-term prospective studies. Nevertheless; cardiac symptoms like palpitations and chest pain were reported in patients with PCS [147]. Of note, silent and progressive cardiac injury in the course of SARS-CoV-2 infection could contribute to the progression of cardiovascular complications, including heart failure, following complete recovery [147]. Persistent cardiac injury was observed in 78% of 100 recovered COVID-19 survivors after 3 months, regardless of initial COVID-19 severity [147].

Furthermore, persistent endothelial dysfunction (ED) in patients with PCS is correlated with the initial severity of SARS-CoV-2 infection [142]. A cohort, case-controlled study comprised of 133 recovered COVID-19 patients 3 months from previous infection compared with matched 133 controls illustrated that flow-mediated dilatation was reduced in recovered COVID-19 patients compared to the controls (*P* < 0.001) [142]. After controlling for major confounders, COVID-19 was identified as an independent risk factor for the development of ED [142]. Similarly, a prospective study involving 70 COVID-19 patients 3 months after infection showed that markers of ED were evident, suggesting that ED in COVID-19 survivors could be implicated in cardiovascular complications in recovered COVID-19 patients [148].

In this state, ED and cardiovascular complications in recovered COVID-19 patients may increase the risk of development of new-onset systemic hypertension [149]. A study of 153 post-COVID-19 patients revealed that both systolic and diastolic blood pressures were higher at post-COVID-19 than at the time of admission [149]. Over-activation of RAS with reduction of Ang1-7, ED, and coagulopathy could be the major causes for the development of hypertension in patients with PCS [149]. Besides, ED and pulmonary thromboembolic disorders may increase the risk of the development of pulmonary hypertension [150].

**Fig. 7:**
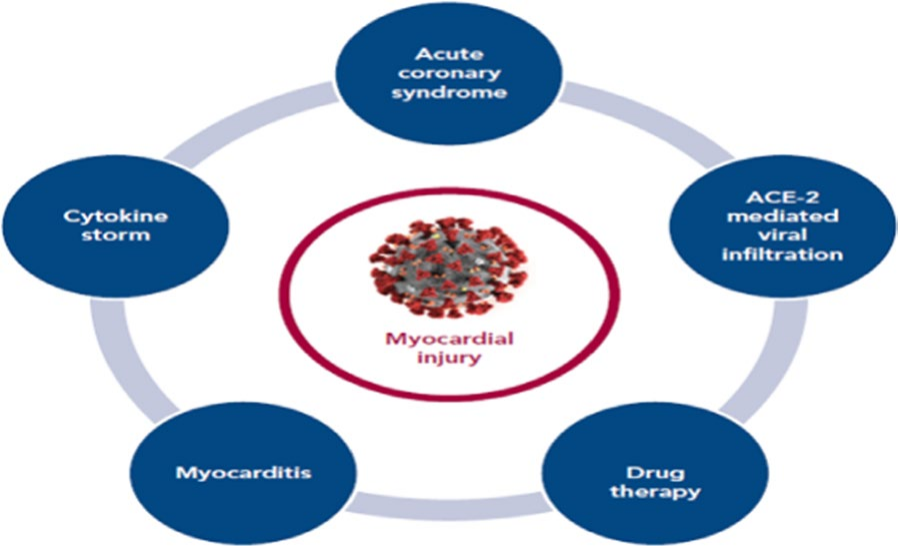
Post-COVID syndrome and cardiovascular complications

These observations indicate that cardiovascular complications in patients with PCS are linked with the development of cardiac injury and new onset hypertension (Fig. 7).

### Renal complications

Acute kidney injury (AKI) may develop in 5% of hospitalized COVID-19 patients and up to 31% of severely ill COVID-19 patients with mechanical injury in acute SARS-CoV-2 infection [151, 152]. Huang et al. in a cohort study disclosed that 13% of recovered COVID-19 patients developed a significant reduction in the estimated glomerular filtration rate at 6 months after acute SARS-CoV-2 infection when renal function was normal during the initial acute phase [100]. AKI in acute COVID-19 increases the risk of developing AKI within 3 months of the COVID-19 [153].

Prolonged inflammatory reactions and thrombotic disorders in COVID-19 survivors increase the risk of developing renal impairments in the high-risk group [153]. Gu et al.'s cohort study found that AKI in acute COVID-19 was related to the reduction of kidney function one year after the acute phase of COVID-19 [154]. As a result, intensive kidney function care during acute COVID-19 may prevent renal impairments in COVID-19 survivors [154].

**Fig. 8:**
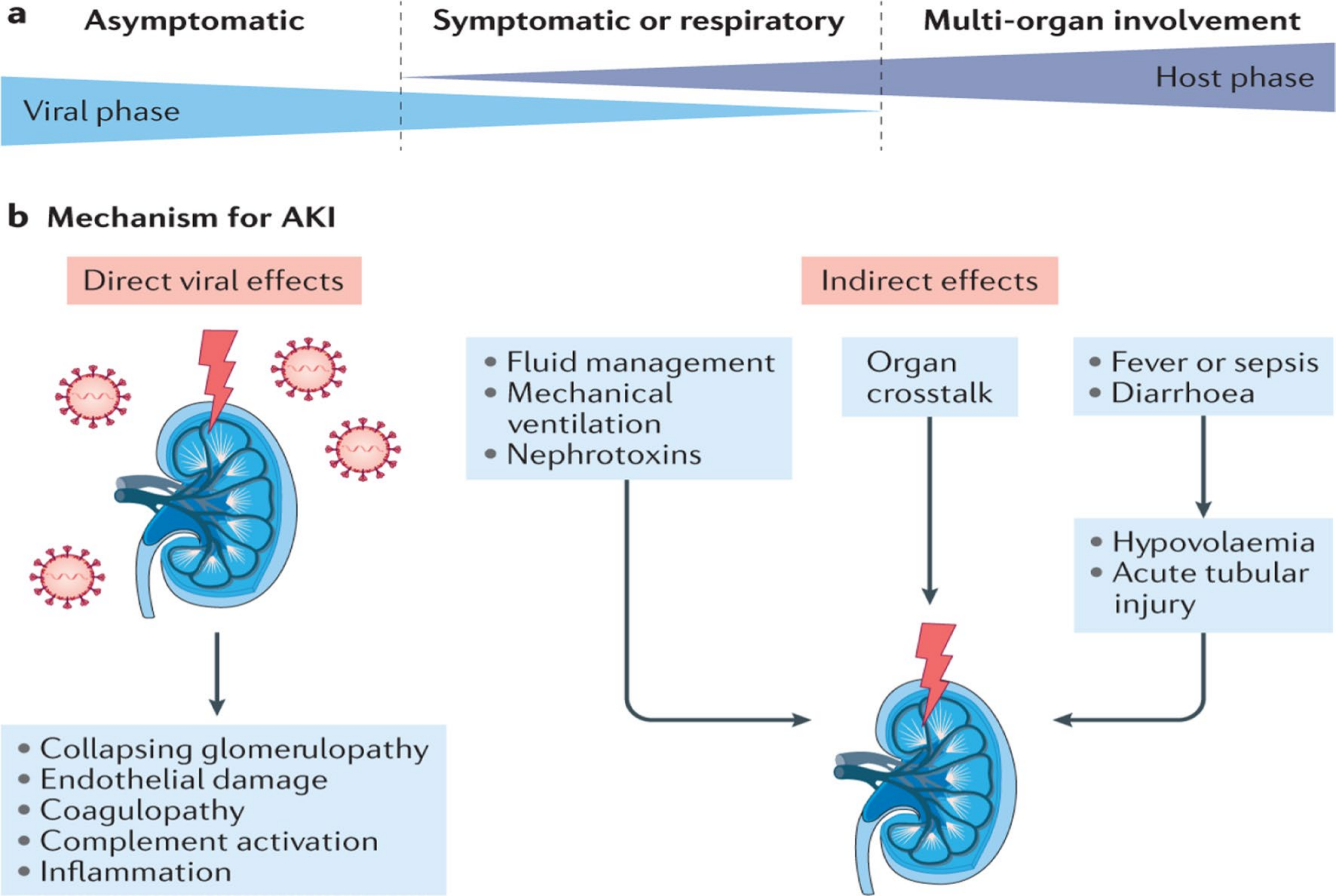
Post-COVID syndrome and renal complications

In brief, the direct cytopathic effect of SARS-CoV-2 infection or indirect effects of COVID-19 may lead to the progression of AKI (Fig. 8).

### Gastrointestinal complications

In patients with PCS, there are different hepatobiliary and gastrointestinal (GIT) disorders, including abdominal pain, vomiting, nausea, diarrhea, and poor appetite due to persistent GIT inflammation [155]. Changes in gut microbiota with the development of dysbiosis by SARS-CoV-2 infection may increase the risk of the development of systemic inflammation and pulmonary dysfunction by the gut-lung axis [155]. GIT symptoms may be observed in COVID-19 survivors in about 84% of cases due to prolonged intestinal inflammation, dysbiosis, and down-regulation of intestinal ACE2 [156]. Latent intestinal inflammation may affect the liver, lungs, and brain via the gut-liver axis, gut-lung axis, and gut-brain axis, respectively [156]. These findings propose that patients with PCS had many symptoms due to GIT disorders induced by the initial SARS-CoV-2 infection. Systemic inflammatory disorders in PCS might be due to persistent SARS-CoV-2 infection in GIT and associated dysbiosis and intestinal inflammation that persist up to 30 days after COVID-19 recovery [107].

**Fig. 9:**
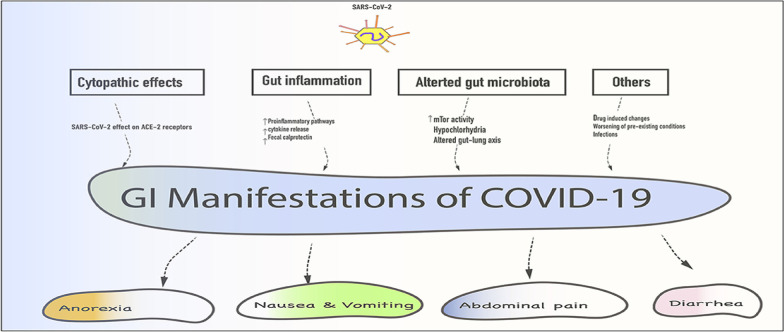
Post-COVID syndrome and gastrointestinal complications

Thus, direct cytopathic effects of SARS-CoV-2 infection with associated gut inflammation and alteration of gut microbiota may lead to GIT manifestation in COVID-19 patients. These changes may induce long-term inflammatory changes with the induction of PCS (Fig. 9).

### Endocrine complications

Different endocrine disorders are addressed with the development of PCS. Diabetic ketoacidosis was reported in non-diabetic COVID-19 survivors within months of recovery from acute SARS-CoV-2 infection [157]. Furthermore, overt thyrotoxicosis, thyroiditis, and Gravis disease have been reported in recovered COVID-19 patients within weeks [158, 159]. In male COVID-19 patients, pituitary–testicular axis dysfunction was reported in recovered patients [160]. In a retrospective study, 143 COVID-19 patients were evaluated at 77 days following disease onset; a low testosterone level was detected in 28.7% of them [160]. Pituitary dysfunction has been observed after acute SARS-CoV-2 infection [161]. A case-controlled study comprised of 43 COVID-19 patients compared to 11 healthy controls illustrated that 46.5% had inadequate growth hormone response and 9.3% had low cortisol response [161]. As well, there was significant elevation in prolactin and thyroid stimulating hormone in 4.6% and 9.3% of recovered COVID-19 patients, respectively [161]. These verdicts advocate that recovered COVID-19 patients had pituitary dysfunction, mainly in the pituitary-adrenal axis and growth hormone response. Interestingly, a case-series of post-COVID hypothalamic pituitary adrenal axis dysfunction was demonstrated as post-COVID-19 perinatal depression and other neuropsychiatric manifestations [162]. This disorder may be the potential cause of post-partum psychosis in recovered pregnant women from COVID-19 [162].

Endocrinopathies in patients with PCS may be due to long-term inflammatory and immunological disturbances and direct viral injury [163]. Pre-diabetic patients may first become overt and apparent during acute SARS-CoV-2 infection and post-COVID-19 with the development of diabetic ketoacidosis due to ACE2-mediated pancreatic injury [164]. PCS has been linked to increased peripheral insulin resistance as well as decreased insulin production from pancreatic-β cells [165]. The prolonged effects of COVID-19 may exacerbate microvascular dysfunction, ED, sarcopenia, and cardiovascular complications with the induction of PCS in diabetic patients [165].

**Fig. 10:**
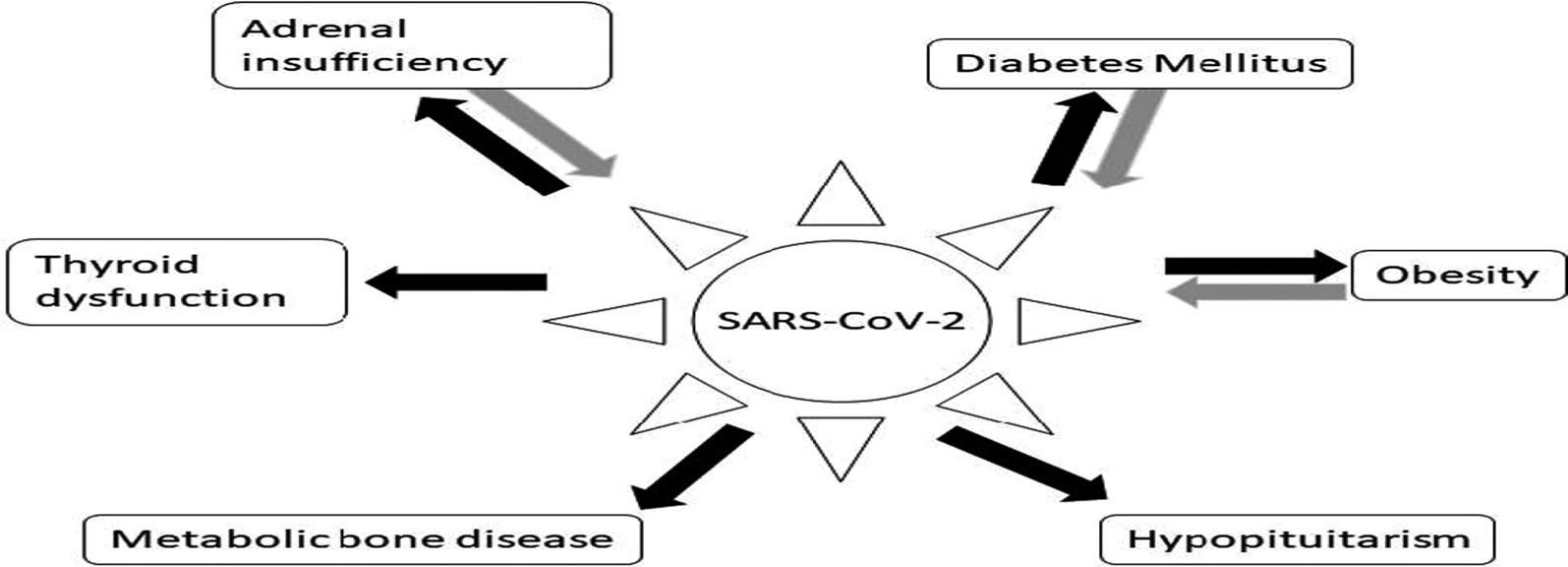
Post-COVID syndrome and endocrine complications

These observations indicate that initial endocrine disorders in acute SARS-CoV-2 infection may persist and contribute to the development of PCS in COVID-19 survivors (Fig. 10).

### Thromboembolic and hematological complications

In PCS patients, there are noteworthy thromboembolic disorders with an incidence of less than 5%. As well, omitting thrombo-prophylaxis in post-COVID-19 patients may increase the risk of thrombosis with the development of cardiac thrombus and ischemic stroke [166]. The median period for the development of thromboembolic complications in PCS patients was 23 days [167]. A case-report study by Boudhabhay et al. suggested that thrombotic microangiopathy can play a crucial role in the pathophysiology of complement-mediated multi-system inflammatory syndrome in PCS patients [168].

**Fig. 11:**
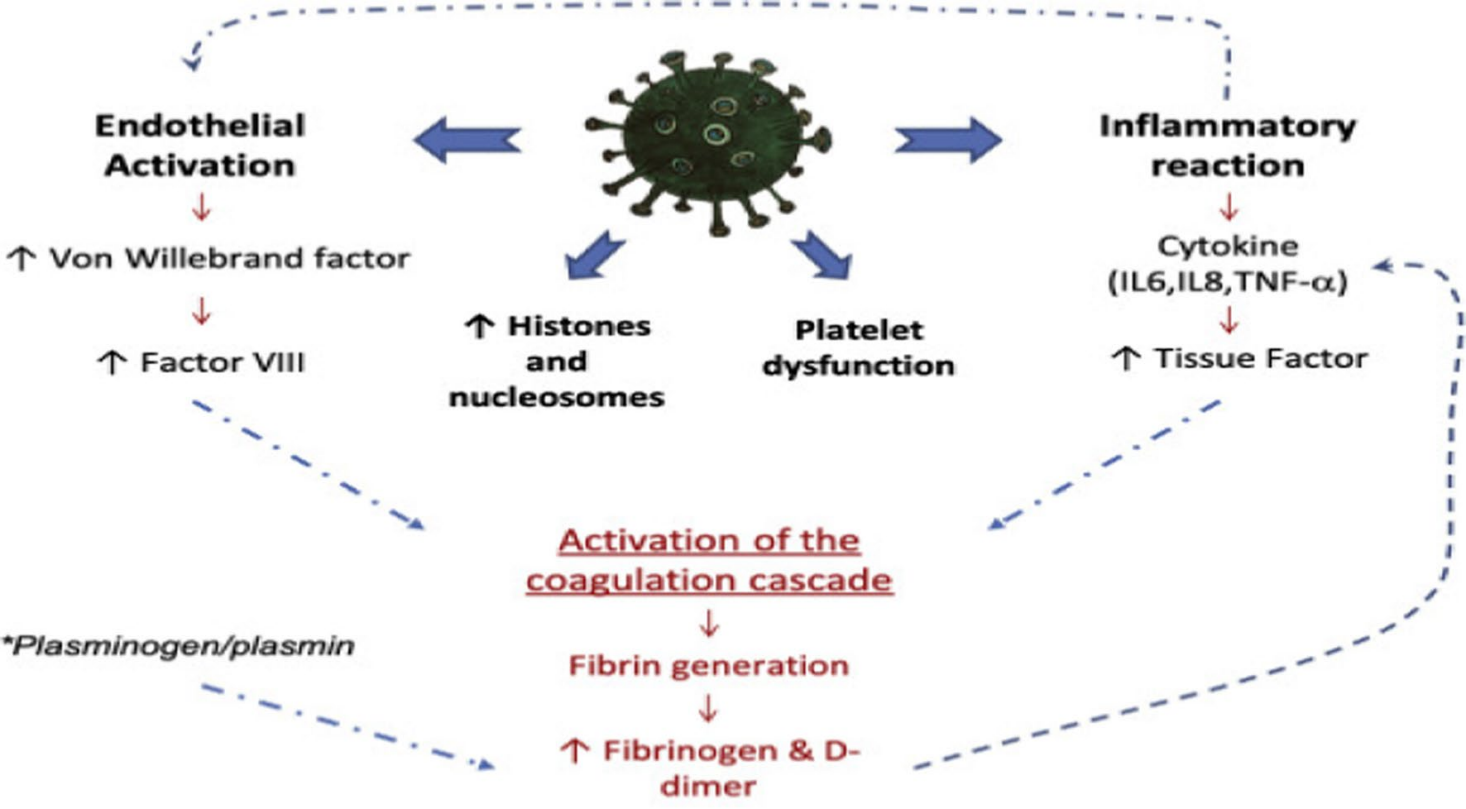
Post-COVID syndrome and thromboembolic complications

The possible mechanisms for thromboembolic complications in PCS patients could be ED, complement activation, development of neutrophil extracellular traps, platelet activation, platelet-neutrophil interactions, hypoxia, and exaggerated pro-inflammatory response [140]. These pathophysiological changes are similar to those seen in thrombotic microangiopathic syndrome in COVID-19 patients [169]. Remarkably, unlike other disorders and complications reported in PCS patients, thromboembolic complications in PCS patients are related to the hyperinflammatory state's severity and duration in the initial acute SARS-CoV-2 infection [140] (Fig. 11).

### Post-COVID syndrome and mast cell activation syndrome

PCS has been hypothesized to be mediated by hyperinflammation and MCAS [170]. Deregulated release of inflammatory mediators in MCAS produces extraordinary symptoms, as evident in patients with PCS [171]. MCAS was first reported in 2007, characterized by allergic and inflammatory disorders with a prevalence of 17% [171]. Dysfunction in the behavior of mast cells following psychological or physical stress may lead to an abnormal release of inflammatory cytokines [170]. In PCS, the interactions of induced stressors may activate mast cell genes by SARS-CoV-2 infection, resulting in abnormal control of mast cell activations [172]. In SARS-CoV-2 infection, activation of toll-like receptors (TLRs) on the immune cells leads to the development of autoantibodies, which may activate mast cells through interaction with immunoglobulin receptors on the mast cells [173].

The key source of various inflammatory cytokines in COVID-19 is mast cells, which are activated by SARS-CoV-2 to release pro-inflammatory cytokines and contribute to the development of pulmonary cytokine storms and other COVID-19 pathologies [174]. Mast cells express ACE2 and can produce vasoconstrictor leukotrienes (LTs) and modulate lung RAS [175]. In general, mast cells are located in the perivascular space of the lungs where they undergo maturation under the effect of micro-environmental factors, causing the induction of hyper-responsiveness [176]. Mast cells are stimulated by allergens cross-linking with IgE Fc epsilon receptor type 1 and non-IgE stimuli through G-protein X2 receptor (GPRX2) by different neuropeptides like substance P and neurotensin [177]. The stimulation of mast cells results in the release of many inflammatory mediators, including platelet activating factor (PAF), histamine, heparin, tryptase, prostaglandins (PG), LTs, and chemokines such as IL-1β and IL-6 [178]. Of note, the release of substance P from immune cells in SARS-CoV-2 infection is augmented [179] as well; a vasoactive peptide storm caused by the increasing release of substance P and neurotensin plays a critical role in the induction of vascular permeability and lung inflammation [180]. As well, SARS-CoV-2 through its domain protein called PSD-95/Dlg/ZO-1 (PDZ), which is found in protein E and N, can activate mast cell GPRX2, resulting in mast cell activation [181]. In this state, both substance P and neurotensin with activation of mast cell GPRX2, which are induced by SARS-CoV-2 infection, could be the possible cause for the development of MCAS in COVID-19.

Interestingly, IL‐6 has been shown to increase MC proliferation and induce a more reactive phenotype providing a possible link between elevated IL‐6 levels and MCAS in PCS. While it remains unclear if MC activation is causative in PCS or simply a consequence, larger longitudinal studies to validate our findings and assess the natural history are critical. Importantly, our findings highlight MCs as potential therapeutic targets for patients with PCS, which could be targeted with agents that reduce MC‐derived mediators, engage inhibitory receptors, or attenuate inflammation [182]. Analogous to respiratory function, it cannot be excluded that the cytokine storm accompanying SARS-CoV-2 infection and MCAS during COVID-19 may indirectly affect female reproductive function, especially in more severe cases [183]. Neutralization of upstream histamine, a major mediator derived from MCs, inhibits the nuclear translocation of NF-*κ*B, thereby preventing the release of the proinflammatory cytokines interleukin (IL-1*β*, TNF-*α*, IL-6, and IL-10). Despite the fact that COVID-19 hyperinflammation and post-COVID-19 illness may be rooted in MCAS, the available clinical data do not provide grounds for treating this mechanism as a significant threat to female reproductive functions, including pregnancy [183]. Understandably, severe COVID-19 reduces female fertility and has been associated with impaired fetal growth during pregnancy, an approximate twofold increased risk of stillbirth, threefold increased risk of preterm birth (likely influenced by iatrogenic deliveries) and prematurity-related worse perinatal outcomes [182, 183]. It should be noted that the pathophysiological spectrum of MCAS is extremely broad and heterogeneous. SARS-CoV-2 infection induced epigenetic mechanisms may explain the increasing incidence of MCAS in PCS. Specific research in the PCS patients with MCAS may related to SARS-CoV-2 infection has been performed to date. Data continue to accumulate, including those regarding the long-term influence of COVID-19 on the course of both preexisting and SARS-CoV-2-induced MCAS. It is noteworthy that PCS with high residual cardiovascular risk and persistence of blood chemistry of inflammation and procoagulative state resembles many aspects of MCAS. For example, in both pathologies, NLRP3 inflammasome may be crucial in the consolidation of coagulation disorders.Further studies are needed to determine whether MCAS-related clotting disorders (e.g., microcoagulopathy) are subjected to long-term modulation by SARS-CoV-2 infection with possible consequences on female reproductive function [182, 183].

In addition to the induced pulmonary inflammation by SARS-CoV-2-induced mast cell activation, mast cell-derived inflammatory and vasoactive mediators can cause derangement of the BBB with the progression of brain fog [184]. In PCS, cognitive dysfunction and fatigue are mainly observed and are similar to those experienced by patients following cancer chemotherapy known as chembrain or chemofog, as well as in patients with ME/CFS or MCAS [185]. Brain fog in COVID-19 may be related to mast cell-induced neuroinflammation through activation of microglia [185]. Development of brain fog in COVID-19 could be the potential cause of persistent neuropsychiatric symptoms in patients with PCS [186]. These observations point out that MCAS may lead to deleterious central and peripheral effects in patients with acute COVID-19 and contribute to the progression of PCS.

It has been shown that the development of MCAS during the course of SARS-CoV-2 infection is correlated with COVID-19 severity and the development of PCS [18]. An observational study illustrated that cytokine storm in patients with severe COVID-19 may be rooted in the advancement of MCAS by SARS-CoV-2 infection [18]. Therefore, the development of MCAS in SARS-CoV-2 infection is linked with poor outcome in severely affected COVID-19 patients, and lung biopsies of COVID-19 patients have large numbers of activated mast cells compared with healthy controls [187]. Schofield reported a case study of POTS linked with the development of MCAS in women with COVID-19 [94]. Of note, MCAS is augmented in PCS due to the activation of mast cells by SARS-CoV-2 through different mechanisms [188].

**Fig. 12:**
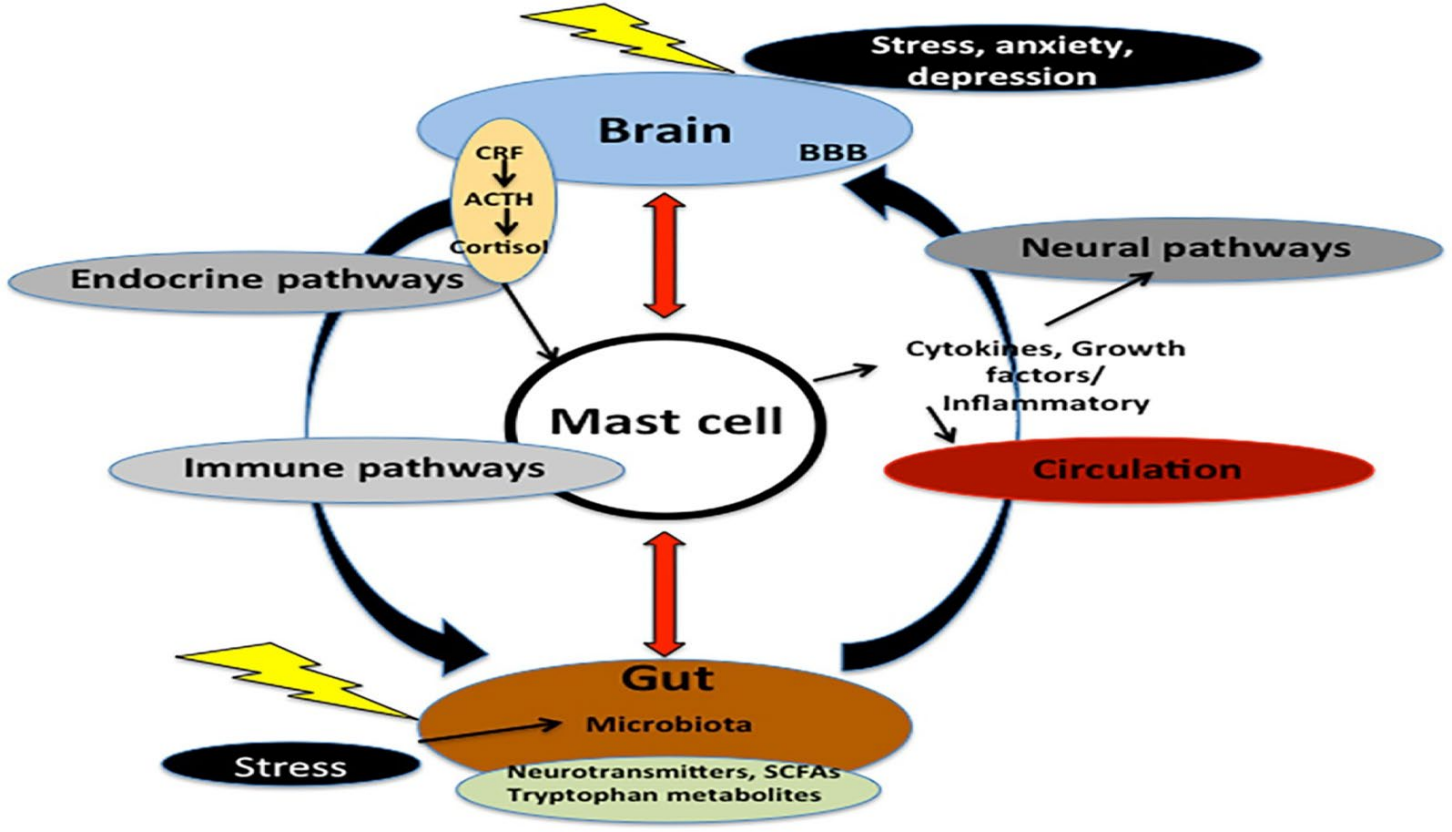
Role of mast cell activation syndrome in the development of Post-COVID syndrome: Stressful conditions during SARS-CoV-2 infection increase the release of corticotropic releasing factor (CRF), adrenocorticotropic hormone (ACTH), and cortisol. Besides, gut stress induces alteration of the gut microbiota, which affects the release of neurotransmitters, tryptophan metabolism, and the release of short-chain free fatty acids (SCFAs). These changes induce activation of mast cells, which releases inflammatory cytokines. Thus, mast cells link the brain and gut through neuronal, immune, and endocrine pathways

Therefore, there are close interactions between SARS-CoV-2 infection and MCAS with the progression of PCS due to the prolonged inflammatory status (Fig. 12).

### Treatment of MCAS in Post-COVID syndrome

In general, MCAS is treated by antihistamines (H1 and H2 blockers), inhibition of synthesis of mediators (zileuton and aspirin), inhibition of mediator release (Na-cromoglycate), and inhibition of degranulation of mast cells by anti-IgE [21].

Famotidine acts as an inverse agonist to inhibit the generation of cAMP or full antagonist to inhibit H2 receptors, thereby reducing endothelial permeability and endothelial dysfunction in Coved-19 [189]. Blocking of H2 receptors and induction of synthesis of cAMP by famotidine can decrease the pathological effects of histamine and mast cell cytokine release, respectively [189]. Therefore, famotidine could be effective against SARS-CoV-2 infection in the acute phase and relieve symptoms of MCAS in patients with PCS.

Zileuton, a 5-lipoxygenase inhibitor, inhibits LT synthesis, preventing hyperinflammation in COVID-19 patients and possibly alleviating MCAS symptoms [4].

Regarding the role of antihistamines in the management of MCAS, H1 blockers alone are not effective in treating symptoms of MCAS, thus H4 blockers could have a promising effect in this state [190]. H4R mediates mast cell activation for migration and release of pro-inflammatory cytokines through mitogen-activated protein kinase (MAPK) [191]. Activation of H4R results in recruitment of mast cells and migration of eosinophils with activation of the immune response and inflammation via activation of T cells and dendritic cells [192]. A clinical trial for the effectiveness of H4R blocker JNJ39758979 showed a notable effect in the management of different allergic disorders [193].

Of note, perturbations of T cells in PCS could be mediated by histamine-dependent mechanisms, so antihistamines could be effective in the management of PCS [194]. Therefore, use of antihistamines in PCS may reduce T cell perturbations and the development of hyperinflammation states [194]. Thus, these observations suggest that antihistamines could be of therapeutic benefit against PCS and associated MCAS.

In addition, mast cell stabilizers like Na-cromoglycate, clarithromycin, and hydrocortisone have important roles in preventing the release of histamine and pro-inflammatory cytokines [195]. Therefore, mast cell stabilizers might be effective in a dual role against PCS and linked MCAS [195].

Finally, treatments that powerfully trigger the immune system, like vaccines, must be given with strong caution as they may exacerbate symptoms of MCAS [196]. However, patients with MCAS or mastocytosis, even those with an anaphylactic history, can be vaccinated safely with COVID-19 vaccines [197].

Due to the uncontrolled release of TGF-β1 by a prolonged inflammatory state [198], pulmonary fibrosis is regarded as the main final fatal complication in PCS patients due to the uncontrolled release of TGF-β1 by a prolonged inflammatory state [198]. Therefore, TGF-β1 inhibitors like triptolide, azithromycin, and vitamin D could be effective in the attenuation of PCPF [199]. Of interest, pirfenidone, a TGF-β1 inhibitor that acts as an anti-fibrotic agent together with anti-inflammatory drugs, may reduce the risk of development of PCPF in COVID-19 survivors [200]. A case report study observed that pirfenidone was effective in preventing the development of PCPF in 40-year-old women who presented with COVID-19 pneumonia [201]. However, TGF-β1 has suppressive effects on mast cell activation both in vivo and in vitro [202]. Therefore, specific inhibitors of mast cells could be effective in the attenuation of the progression of PCS. Notably, butyrate inhibits mast cell activation by inhibiting FcR1-mediated signalling [203], ruxolitinib inhibits mast cell activation by suppressing the JAK1/JAK2 pathway [204], and monoclonal antibody against Siglec-8 inhibits airway inflammation via an IgE-independent pathway [205]. Thus, the inhibitors of mast cell activation could be effective in preventing the MCAS-induced progression of PCS in COVID-19 survivors.

## Conclusions

PCS can develop within 3 months of acute COVID-19, but it can also develop after mild or asymptomatic COVID-19.The underlying pathophysiology of PCS is still unidentified, though immune dysregulation, persistence of inflammatory reactions, autoimmune mimicry, and reactivation of pathogens together with host microbiome alterations may contribute to the development of PCS. PCS may progress in association with the development of mast cell activation syndrome (MCAS). The emergence of MCAS during the course of SARS-CoV-2 infection is linked to the severity of COVID-19 and the emergence of PCS. Therefore, the use of antihistamines, inhibition of synthesis/release of mediators, and suppression of mast cell degranulation by anti-IgE may reduce MCAS-induced development of PCS. Perturbations of T cells in PCS could be mediated by histamine-dependent mechanisms, so antihistamines could be effective in the management of PCS. Taken together, the early recognition and treatment of MCAS in PCS patients may reduce systemic complications and long-term organ dysfunction.

## Data Availability

All collected data are discussed in the manuscript.
